# Supplementary material for: The interactome of intact mitochondria by cross-linking mass spectrometry provides evidence for coexisting respiratory supercomplexes
Source: Mol Cell Proteomics. 2017 Dec 8;17(2):216–32. doi: 10.1074/mcp.RA117.000470 (PMC5795388; doi:10.1074/mcp.RA117.000470)
Supplement: Supplemental Data [file supp_RA117.000470_133922_0_supp_23978_fzffwf.zip › spectra_annotation/mito_DR_spectra_annotation/353-1-1-1-16-1.pdf]

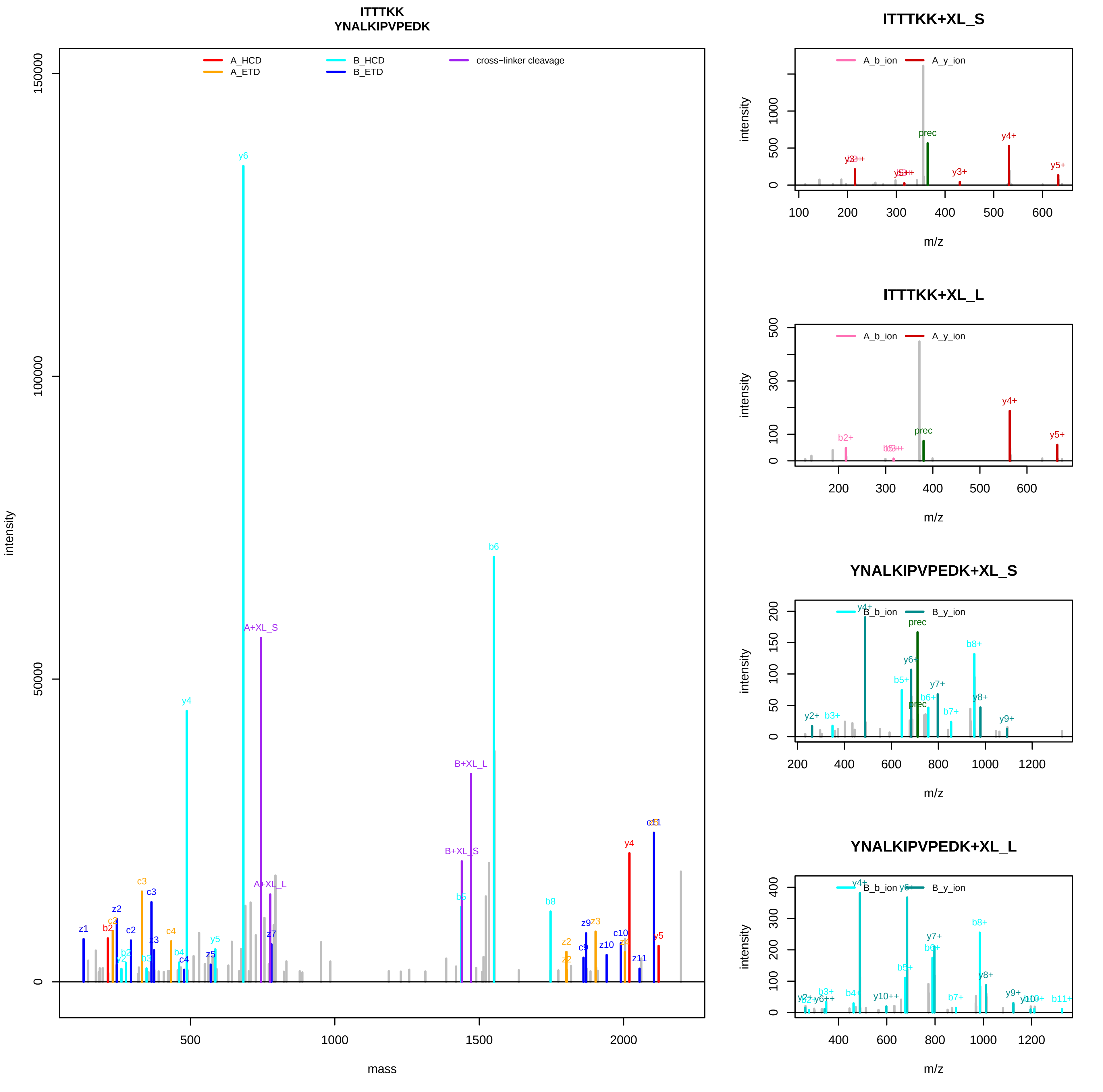
ITTTKK
YNALKIPVPEDK
A_HCD
A_ETD
B_HCD
B_ETD
cross−linker cleavage
intensity
mass
0
50000
100000
150000
500
1000
1500
2000
y6
b6
A+XL_S
y4
B+XL_L
B+XL_S
A+XL_L
y4
c11
b8
c3
c3
y5
b5
y5
z7
c10
z9
z3
c9
z10
z11
z1
b2
z2
c2
c4
b4
z5
ITTTKK+XL_S
A_b_ion
A_y_ion
prec
y4+
y5+
y3+
intensity
m/z
0
500
1000
100
200
300
400
500
600
ITTTKK+XL_L
A_b_ion
A_y_ion
b2+
prec
y4+
y5+
0
100
300
500
200
300
400
500
600
intensity
m/z
YNALKIPVPEDK+XL_S
B_b_ion
B_y_ion
y4+
prec
b8+
y6+
b5+
y7+
b6+
y8+
b7+
y2+
b3+
y9+
0
50
100
150
200
200
400
600
800
1000
1200
intensity
m/z
YNALKIPVPEDK+XL_L
B_b_ion
B_y_ion
y4+
y6+
b8+
y7+
b6+
b5+
y8+
b4+
b3+
y10++
b7+
y9+
b11+
0
100
200
300
400
400
600
800
1000
1200
intensity
m/z
